# An overview of national-level excise taxes on e-cigarettes across the world

**DOI:** 10.18332/tid/171353

**Published:** 2023-10-10

**Authors:** Qinghua Nian, Kevin Welding, Zheng Dai

**Affiliations:** 1 Institute for Global Tobacco Control, Department of Health, Behavior and Society, Johns Hopkins Bloomberg School of Public Health, Johns Hopkins University, Baltimore, United States; 2 Department of Economics, The George Washington University, Washington, United States

**Keywords:** nicotine, e-cigarette, excise tax

**Dear Editor,**

E-cigarettes have become increasingly popular globally, with sales reaching US$ 21.2 billion in 2020^1^. These devices vaporize a liquid solution that usually contains nicotine, flavorings, and other chemicals^2^. We collected e-cigarette excise tax policies in regard to the type of excise tax, the tax base, and any relevant details that were available online as of July 2022 from the Institute for Global Tobacco Control’s Policy Scan^3^, World Bank reports^4^, European Policy Information Center^5^, and Tax Data Center^6^.

Three different options for imposing excise taxes on e-cigarettes were found: 1) a specific tax, 2) an ad valorem tax (VAT), and 3) a combination of these two. In all, 39 countries have enacted e-cigarette excise taxes. Italy was the first to levy a tax in 2014. Among the countries that impose e-cigarette excise taxes, 32 countries (82.1%) solely levy a specific tax, five countries (12.8%) solely an VAT, and two countries (5.1%) levy both specific and VAT (Table 1). Among the 34 countries levying specific taxes, 26 countries (76.5%) had a fixed rate based on liquid volume, cartridges unit, device unit, or nicotine concentration (mg/mL); and eight countries (23.5%) had a variable rate based on nicotine concentration level, nicotine salt in liquid, or device type. Among countries levying a fixed rate specific tax on liquid volume, the rate ranges from US$ 0.01 per mL in Latvia to US$1.38 per mL in South Korea; and 20 countries (90.9%) use a rate <US$ 0.5 per mL^7^. Among countries levying a variable rate specific tax on nicotine concentration and formulation of liquid, Sweden nearly doubles the tax rate for e-cigarettes with nicotine concentration ≥15 mg/mL (0.39 US$ 0.39 per mL) compared to those with <15 mg/mL (US$ 0.20 per mL) and Philippines exerts a tax rate on e-cigarettes with salt liquid (US$ 0.76 per mL) that is nearly eight times the tax rate on e-cigarettes with freebase liquid (US$ 0.10 per mL). Regarding device type, Israel and Indonesia levy much higher tax rates on closed systems (i.e. disposable devices and disposable pods/cartridges) than open systems (360% vs 270% of the sales price, US$ 0.41 vs US$ 0.03 per mL, respectively). Among the seven countries that levy a VAT, the rate ranges from 30% of the sales price in Nepal to 360% of the sales price in Israel.

**Table 1:** National-level e-cigarette excise tax policy by country, as of July 2022 (N=39)

*Tax type*		Country***	*Tax based on*	*Tax rate range*
Specific (n=34)	Fixed rate (n=26)	Albania, Azerbaijan, Croatia, Cyprus, Denmark, Egypt*, Estonia, Finland, Germany, Greece, Guam, Hungary, Kazakhstan, Kyrgyzstan, Latvia, Lithuania, Montenegro, North Macedonia, Norway, Poland, Romania, Russia, Serbia, Slovenia, South Korea	Volume of liquid (per mL) (n=25)	US$ 0.01–1.38
		Kenya, South Korea	Cartridge (per unit) (n=2)	US$ 0.019–0.021
		Kenya, Russia	Device (per unit) (n=2)	US$ 0.03–1.00
		Latvia	Nicotine concentration (mg/mL) (n=1)	US$ 0.005
	Variable rate (n=8)	Italy, Portugal, Sweden, Ukraine, Uzbekistan	Volume of liquid (per mL) by nicotine concentration level (n=5) **	Volume of liquid with nicotine (per mL): US$ 0.03–0.39 Volume of liquid without nicotine (per mL): US$ 0.00–0.09
		Philippines	Volume of liquid (per mL) by nicotine salt (n=1)	Volume of nicotine salt liquid (per mL): US$ 0.76 Volume of freebase liquid (per mL): US$ 0.10
		Indonesia, Israel*	Volume of liquid (per mL) by device type (n=2)	Indonesia Volume of liquid (per mL) of closed device: US$ 0.41 Volume of liquid (per mL) of open device: US$ 0.03 Israel Volume of liquid (per mL) of refillable e-cigarettes: US$ 3.31
Ad valorem (n=7)		Bahrain, Ecuador, Egypt*, Israel*, Jordan, Nepal, Saudi Arabia	Sales price (n=5)	30%-360% of the sale price

* Egypt and Israel impose both a specific and an ad valorem tax on e-cigarettes.

** Portugal, Sweden, Ukraine and Uzbekistan impose excise taxes only on nicotine-containing liquids; Italy imposes the excise tax on liquids with nicotine (US$ 0.14/mL) and without nicotine (US$ 0.09/mL).

*** Countries and jurisdictions started levying excise taxes on e-cigarettes in: 2014 – Italy; 2015 – Kazakhstan and Kenya; 2016 – Latvia, Romania and Slovenia; 2017 – Croatia, Cyprus, Finland, Greece, Hungary, Montenegro and Russia; 2018 – Bahrain, Estonia, Indonesia and Sweden; 2019 – Albania, Azerbaijan, Jordan, Lithuania, North Macedonia and Saudi Arabia; 2020 – Ecuador, Egypt, Kyrgyzstan, Philippines and Uzbekistan; 2021 – Israel, Norway, Poland, Portugal and Ukraine; 2022 – Denmark and Germany; unknown – Guam, Nepal, Serbia and South Korea.

We have presented the landscape of e-cigarette excise tax policies worldwide, which may provide policy options for taxing e-cigarettes. Among 39 countries that levy excise tax on e-cigarettes, the majority (n=25; 64.1%) impose a specific tax with a fixed rate based on liquid volume. The tax rate varies greatly across countries. Some countries consider device and liquid characteristics when imposing taxes on e-cigarettes and apply higher tax rates on closed systems, nicotine salt liquids, and liquids with higher nicotine concentrations. One limitation is that sub-national (e.g. United States) and e-cigarette tax policies reported after July 2022 are not included.

## Data Availability

Data sharing is not applicable to this article as no new data were created.
